# Corneal confocal microscopic characteristics of acute angle-closure crisis

**DOI:** 10.1186/s12886-022-02253-w

**Published:** 2022-01-11

**Authors:** Weiwei Wang, Xin Yang, Qian Yao, Qianqian Xu, Wenting Liu, Jianrong Liu

**Affiliations:** Shaanxi Eye Hospital, Xi’an People’s Hospital (Xi’an Fourth Hospital), Affiliated Guangren Hospital, School of Medicine, Xi’an Jiaotong University, Xi’an, 710004 China

**Keywords:** Acute angle-closure crisis, Cornea, Confocal microscopy

## Abstract

**Background:**

To investigate characteristics of the acute angle-closure crisis (AACC) and fellow eyes using confocal microscopy.

**Methods:**

Unilateral AACC patients hospitalized at the Xi’an People’s Hospital from October 2017 to October 2020 were recruited in this cross-sectional study. Age-matched participants scheduled for cataract surgery were enrolled as a healthy control group. Corneal epithelial cells, subepithelial nerve fiber plexus, stromal cells, and endothelial cells were examined by confocal and specular microscopy.

**Results:**

This study enrolled 41 unilateral AACC patients (82 eyes) and 20 healthy controls (40 eyes). Confocal microscopy revealed that the corneal nerve fiber density, corneal nerve branch density and corneal nerve fiber length were reduced significantly in AACC eyes. The stromal cells were swollen and the size of the endothelial cells was uneven with the deposition of punctate high-reflective keratic precipitate on the surface. In severe cases, the cell volume was enlarged, deformed, and fused. The corneal subepithelial nerve fiber, stromal layer, and endothelial layer were unremarkable in the fellow eyes, and the density of the endothelial cells was 2601 ± 529 cells/mm^2^, which was higher than 1654 ± 999 cells/mm^2^ in AACC eyes (*P* < 0.001). Corneal edema prevented the examination of 17 eyes using specular microscopy and in only four eyes using confocal microscopy. There were no significant differences in endothelial cell density between confocal and specular microscopy in the AACC eyes (*P* = 0.674) and fellow eyes (*P* = 0.247). The hexagonal cell ratio reduced significantly (*P* < 0.001), and average cell size and coefficient of variation of the endothelial cells increased significantly compared with fellow eyes (*P* < 0.001, *P =* 0.008).

**Conclusions:**

AACC eye showed decreased density and length of corneal subepithelial nerve fiber plexus, activation of stromal cells, increased endothelial cell polymorphism, and decreased density.

## Background

Primary angle-closure glaucoma (PACG) is a form of glaucoma characterized by narrowing or closure of the irido-corneal angle that results in inadequate drainage of the aqueous humor, increased intraocular pressure (IOP), and damage to the optic nerve [1–3]. PACG is one of the main types of glaucoma leading to irreversible blindness in the Asian population [1]. Angle-closure is due to appositional or synechial closure of the irido-corneal angle resulting from anatomical or physiological abnormalities of the eye in primary angle-closure or factors that pull or push the iris forward in case of secondary glaucoma [2, 3].

Angle-closure can result in acute angle-closure crisis (AACC) with a sudden and symptomatic elevation of IOP resulting in a dramatic elevation in IOP accompanied by related symptoms and histopathological changes in the anterior segment of the eye or chronic angle-closure with gradually elevated IOP over a long period that is generally asymptomatic but can eventually lead to damage to the optic nerve and vision impairment or loss [1, 2]. The worldwide prevalence of AACC in adults of 40–80 years of age is 0.5% [4], but its prevalence can reach 3% in Chinese and 3.8% in Inuit [1]. PACG is generally bilateral, although 90% of acute attacks are unilateral by the sudden closure of the irido-corneal angle [5]. It frequently occurs in older women > 50 years of age [1]. During AACC, high IOP leads to severe headache, ophthalmalgia, photophobia, lacrimation, corneal edema, and vision loss in addition to decreased density of corneal endothelial cells, corneal decompensation, and blindness [1, 2, 6]. Since PACG is bilateral, the fellow eye is also at high risk of AACC, with a risk of 50% within 5 years [1].

Specular microscopy should be performed for glaucoma patients to check the endothelial cell density before intraocular surgery, but corneal edema caused by acute IOP elevation often makes it impossible to analyze the endothelial cells [7, 8]. Confocal microscopy is a novel non-invasive corneal imaging examination method [9–11]. Due to high resolution and magnification, it reveals the corneal changes at the cellular level under three-dimensional space and real-time conditions; similar results could be obtained in the cloudy corneal tissue, making it a commonly used tool for the clinical study of corneal lesions [9–14].

Confocal microscopy plays a major role in evaluating keratitis and corneal nerves [9–14], but there is only one study about its use for AACC which focused on Langerhans cells and keratocytes [15]. Our study aimed to investigate corneal epithelial cells, subepithelial nerve fiber plexus, stromal cells, and endothelial cells of the AACC and fellow eyes and healthy controls using confocal microscopy. The results could provide an additional tool for the evaluation of eyes with AACC.

## Methods

### Study design and participants

PACG patients hospitalized at the Department of Ophthalmology in Xi’an People’s Hospital (Xi’an Fourth Hospital) from October 2017 to October 2020 were recruited in this cross-sectional study. The inclusion criterion was fulfilling the diagnostic criteria of unilateral AACC [1]. The exclusion criteria were 1) secondary angle-closure, 2) history of eye surgery, 3) history of other eye diseases except for refractive error and cataract, 4) perimetry showed fixation loss rate > 20%, false positive rate > 15%, and false-negative rate > 15%, or 5) abnormal scotoma in perimetry of fellow eyes. This study was performed in accordance with the Declaration of Helsinki and approved by the Ethics Committee of Xi’an People’s Hospital (Xi’an Fourth Hospital). Written informed consent was obtained from all subjects before participation in the study.

### Data collection

The patient’s age, diseases, and other information were from the hospital’s electronic medical record system.

### Preparation before examination

During AACC, all patients received an intravenous drip of 250 mL of mannitol and carteolol hydrochloride eye drops (China Otsuka Pharmaceutical Co., Ltd. Shanghai, China), brinzolamide eye drops (Alcon, Hunenberg, Switzerland), brimonidine tartrate eye drops (Allergan, Dublin, Ireland), and pilocarpine nitrate eye drops (Bausch & Lomb, Bridgewater, NJ, USA) to reduce IOP and restore the transparency of the cornea and improve the effectiveness and accuracy of the examination. Some patients received oral methazolamide tablets (25 mg, bid, Hangzhou AoyiPollenin Pharmaceutical Co., Ltd., Hangzhou, China) to lower IOP. In the fellow eyes, pilocarpine nitrate eye drops were administered to avoid the occurrence of AACC.

### Routine eye examination

Visual acuity was examined using the standard visual acuity chart (converted into LogMAR visual acuity for statistical analysis). A non-contact tonometer (Canon TX-20, Canon, Tokyo, Japan) was used to measure the IOP. Direct ophthalmoscopy and/or pre-set lens were used to check the fundus. The gonioscope was a Goldmann 2-mirror lens (Ocular Instruments, Bellevue, WA, USA). The perimetry was assessed using a Humphrey 750i Perimeter (Carl Zeiss GmbH, Oberkochen, Germany). Ultrasound biomicroscopy (UBM) was performed using an Aviso system (Quantel Medical, Cournon d’Auvergne, France). The specular microscope was an EM-3000 (Tomey Corp., Nagoya, Japan).

### Confocal microscopy

The cornea module was examined using a confocal microscope (HRT-3/RCM, Heidelberg Engineering Inc., Heidelberg, Germany). The laser wavelength was 670 nm, the magnification was × 800, and the resolution was 1 μm [16]. Before the examination, both eyes were anesthetized with 0.4% oxybuprocaine hydrochloride eye drops (Santen Pharmaceutical Co., Osaka, Japan), and 0.2% carbomer eye drops (Shandong Freda Biotechnology Co., Ltd., Shandong, China) were applied to the surface of the objective lens. The “section” mode was used to scan the cornea layer by layer to observe the morphological characteristics of the tissues and cells in each layer. All procedures were performed by the same specialist with 8 years of experience in corneal confocal microscopy.

Each cornea was scanned in five regions: central, superior, inferior, nasal, and temporal parts of the cornea. The three most representative images were selected for each area, and the field of view for each image was 400 × 400 μm. Fifteen micrographs were selected for each eye, and corneal subbasal nerve plexus was analyzed by ACCMetrics software (University of Manchester). The nerve metrics were 1) corneal nerve fiber density (CNFD; the number of fibers/mm^2^), 2) corneal nerve branch density (CNBD; the number of branch points on the main fibers/mm^2^), 3) corneal nerve fiber length (CNFL; the total length of fiber mm/mm^2^), 4) corneal nerve fiber total branch density (CTBD; the total number of branch points/mm^2^), 5) corneal nerve fiber area (CNFA; the total nerve fiber area mm^2^/mm^2^), 6) corneal nerve fiber width (CNFW; average nerve fiber width mm/mm^2^) [17].

### Statistical analysis

Statistical analysis was performed using SPSS 19.0 (IBM Corp., Armonk, NY, USA). Continuous data are displayed as means ± standard deviation, and categorical data are displayed as n (%). Visual acuity was converted into LogMAR visual acuity for statistical analysis. A paired sample t-test was used to compare the differences of LogMAR visual acuity, IOP, and endothelial cell density between AACC eye and fellow eye, and the age between AACC patients and healthy controls, and the corneal endothelial cell density between confocal and specular microscopy at the same eyes. *χ*^*2*^ test was used for comparison of the categorical data between AACC patients and healthy controls. The continuous data among AACC eye, fellow eye and healthy control were compared using one-way analysis of variance (ANOVA), and differences between the two groups were analyzed by Fisher’s Least Significant Difference (LSD) test. *P*-values < 0.05 were considered statistically significant.

## Results

### Characteristics of the participants

A total of 41 patients (82 eyes) with unilateral AACC were enrolled. The cohort consisted of 34 (82.9%) females, and the mean age of the patients was 64.6 ± 8.4 years. The time from disease onset to hospital visit was 12.9 ± 8.8 days. Twenty-four (58.5%) right eyes and 17 (41.5%) left eyes were in the AACC phase. Twenty age-matched participants scheduled for cataract surgery were enrolled as a healthy control group. The patients consisted of 16 (80%) females, and the mean age was 64.5 ± 7.0 years. The characteristics of the 41 AACC patients (82 eyes) and 20 healthy controls (40 eyes) are listed in Table 1.

**Table 1:** Characteristics of the patients with acute angle-closure crisis and healthy controls

	Patients (***n*** = 41)	Healthy controls (***n*** = 20)	***P***
Age (years)	64.6 ± 8.4	64.5 ± 7.0	0.969
Sex			
Male	7 (17.1%)	4 (20%)	0.780
Female	34 (82.9%)	16 (80%)	
AACC eye			
Left	17 (41.5%)		
Right	24 (58.5%)		
Hypertension	13 (31.7%)	6 (30%)	0.892
T2DM	2 (4.9%)	1 (5%)	0.984
History of coronary heart disease	1 (2.4%)	–	
Average time from onset to hospitalization (day)	12.9 ± 8.8	–	

The age is displayed as mean ± standard deviation. Other data is displayed as n (%)

*AACC* Acute angle-closure crisis, *T2DM* Type 2 diabetes

### Confocal microscopy

**Fig. 1:**
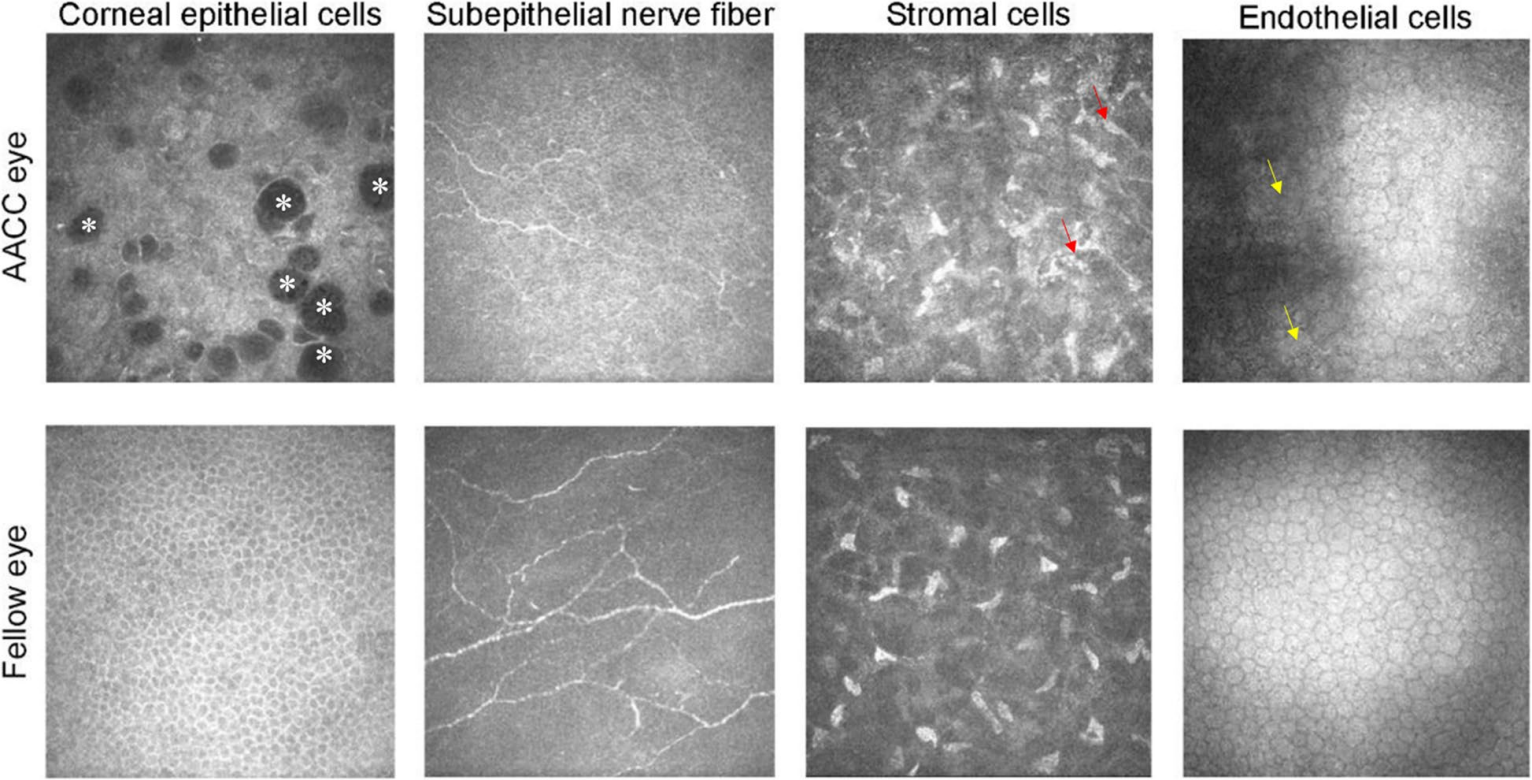
Characteristics of eyes with acute angle-closure crisis (AACC) compared with fellow eyes. Corneal epithelial cells in the AACC eyes varied in size, and the intercellular space was widened. In severe patients, large vacuoles (*) were present in the epithelial cells, which showed a low-density reflective area with distinct boundaries. In the fellow eyes, cells showed regular morphology and arrangement. The subepithelial nerve fiber plexus was reduced in the AACC eyes, and the nerve fiber plexus was normal in the fellow eyes. Swollen stromal cells in the AACC eyes were cross-linked into a network with enhanced reflection (red arrow), and the nuclei of stromal cells in the fellow eyes were visualized against a dark background. The endothelial cell size in the AACC eyes was uneven, and the cell volume was enlarged (yellow arrow), deformed, and fused, and punctate deposition of high-reflective keratic precipitate could be seen on the surfaces of the cells. In the fellow eyes, the regular flat honeycomb hexagonal cells and a high reflection of the cell body were seen

In the AACC eyes, the corneal epithelial cells were swollen, of variable size, and the intercellular space was widened. In severe patients, large vacuoles were present in the epithelial cells, which showed a low-density reflective area with distinct boundaries (Fig. 1). For subepithelial nerve fiber plexus, significant reduction of CNFD, CNBD, and CNFL was found (Table 2, Fig. 1). The stromal layer was in an activated state, showing swollen stromal cells, enhanced reflection, and cross-linkage into a network (Fig. 1). In four eyes, the endothelial layer was blurred, and the cell density could not be calculated. In the other 37 eyes, the endothelial cells were of uneven size, and dark areas were noted among the cells. The surfaces of the cells showed punctate deposition of high-reflective keratic precipitate (Fig. 1). In severe cases, the cell volume was enlarged, the cells were deformed and fused.

**Table 2:** Analysis of corneal nerve plexus by confocal microscopy

	AACC eye (***n*** = 37^**c**^)	Fellow eye (***n*** = 41)	Healthy control (***n*** = 40)	***P***^**#**^
CNFD	7.0 ± 2.1^ab^	15.0 ± 3.4	18.7 ± 6.0	< 0.001
CNBD	10.6 ± 4.7^ab^	25.0 ± 8.8	32.2 ± 17.3	< 0.001
CNFL	9.4 ± 3.1^b^	11.2 ± 2.0	13.0 ± 2.9	0.005
CTBD	32.5 ± 14.4	40.0 ± 15.0	43.7 ± 28.3	0.367
CNFA	0.0060 ± 0.0024	0.0057 ± 0.0014	0.0059 ± 0.0019	0.941
CNFW	0.0235 ± 0.0015	0.0235 ± 0.0013	0.0218 ± 0.0022	0.030

*CNFD* Corneal nerve fiber density (the number of fibers/mm^2^), *CNBD* Corneal nerve branch density (the number of branch points on the main fibers/mm^2^), *CNFL* Corneal nerve fiber length (the total length of fiber mm/mm^2^), *CTBD* Corneal nerve fiber total branch density (the total number of branch points/mm^2^), *CNFA* Corneal nerve fiber area (the total nerve fiber area mm^2^/mm^2^), *CNFW* Corneal nerve fiber width (average nerve fiber width mm/mm^2^)

^a^Comparison between AACC and Fellow eye, ^b^ Comparison between AACC and Healthy control, *P* < 0.05

^c^The data of 37 eyes were analyzed by confocal microscopy

^#^Comparison among AACC, fellow eye and healthy control

In the fellow eyes, corneal epithelial cells showed regular pentagonal or hexagonal shape, with regular morphology and arrangement (Fig. 1). Reduction of CNFD, CNBD, and CNFL was found, but the differences were not significantly compared with the healthy controls (Table 2, Fig. 1). The stromal cells showed a nucleus against a dark background (Fig. 1), but the cytoplasm, cell boundaries, and collagen layer were not detected. The endothelial cells showed a layer of regular flat honeycomb hexagonal cells, and a high reflection of the cell body was seen (Fig. 1). Furthermore, low reflection was noted for the cell boundary and intercellular space, and the cell nuclei were not observed.

There were no significant differences between the AACC eyes and fellow eyes in LogMAR visual acuity and IOP (after applying IOP-lowering drugs). The CNFD and CNBD reduced significantly in the AACC eyes compared with fellow eyes (*P* < 0.001, *P* = 0.005). The density of the endothelial cells in AACC eyes and fellow eyes was 2601 ± 529 cells/mm^2^ and 1654 ± 999 cells/mm^2^ (Table 3). The density of the endothelial cells in AACC and fellow eyes differed significantly (*P* < 0.001) (Tables 3 and 4).

**Table 3:** Endothelial cell density of confocal and specular microscopy

	AACC eye (***n*** = 37^**a**^)	Fellow eye (***n*** = 41)	Healthy control (***n*** = 40)	***P***^**#**^
Vision (LogMAR)	1.4 ± 1.1	0.3 ± 0.2	0.8 ± 0.4	< 0.001
IOP (mmHg)	27.1 ± 15.4	12.4 ± 3.1	13.1 ± 2.9	< 0.001
**Endothelial cell density**				
CM	1654 ± 999	2601 ± 529	2593 ± 256	< 0.001
SM	1982 ± 812^b^	2544 ± 473	2554 ± 224	< 0.001
P*	0.674	0.247	0.941	

*AACC* Acute angle-closure crisis, *IOP* Intraocular pressure, *CM* Confocal microscopy, *SM* Specular microscopy

^#^Comparison among AACC eye, fellow eye and healthy controls

*Comparison between confocal microscopy and specular microscopy

^a^The data of 37 eyes and 24 eyes were analyzed by confocal microscopy and specular microscopy, respectively

^b^Comparison between AACC eye and fellow eye

**Table 4:** Analysis of corneal endothelial cells by specular microscopy

	AACC eye (***n*** = 24^c^)	Fellow eye (***n*** = 41)	Healthy control (***n*** = 40)	***P***^**#**^
Cell density	1982 ± 812^a b^	2544 ± 473	2554 ± 224	< 0.001
Average cell size (μm^2^)	633.09 ± 409.06^a b^	402.70 ± 103.08	399.53 ± 34.47	< 0.001
Coefficient of Variation (%)	45.09 ± 10.94^a b^	39.15 ± 8.71	40.08 ± 6.12	0.023
Hexagonal cell ratio (%)	35.30 ± 11.45^a b^	45.75 ± 8.45	51.85 ± 8.19	< 0.001
Central corneal thickness (μm)	549.40 ± 39.33	530.65 ± 38.18	533.41 ± 31.18	0.158

^#^Comparison among AACC, fellow eye and healthy control

^a^Comparison between AACC and fellow eye, ^b^ Comparison between AACC and healthy control, *P* < 0.05

^c^The data of 24 eyes were analyzed by specular microscopy

### Specular microscopy

In AACC eyes, endothelial cell density and hexagonal cell ratio reduced significantly (Table 4), and average cell size and coefficient of variation of the endothelial cells increased significantly compared with fellow eyes or healthy controls (Table 4). The central corneal thickness in AACC eyes raised in comparison with fellow eyes or healthy controls, but the differences were not significant (*P* = 0.066, *P* = 0.111, Table 4).

### Confocal vs. specular microscopy

As expected, visual acuity and IOP were deteriorated in AACC eyes compared with their fellow eyes (both *P* < 0.001). In AACC, the endothelial cells in 17 eyes could not be analyzed by specular microscopy due to corneal edema, and the density of the endothelial cells in the remaining 24 eyes was 1982 ± 812 cells/mm^2^. The difference in endothelial cell density between fellow eyes and AACC eyes was significant under specular microscopy (*P* = 0.017) (Table 3). There was no significant difference between specular and confocal microscopy in AACC eye (*P* = 0.674) (Table 3). In the fellow eyes, corneal endothelial cell density was 2544 ± 473 cells/mm^2^ under the specular microscopy, with no significant difference compared with 2601 ± 529 cells/mm^2^ under confocal microscopy (*P* = 0.247).

## Discussion

Confocal microscopy can be used to evaluate keratitis and corneal nerves [9–14], but there is no study about its use in eyes with AACC. This study aimed to investigate corneal epithelial cells, subepithelial nerve fiber plexus, stromal cells, and endothelial cells of the AACC and fellow eyes using confocal microscopy. The results suggest that AACC can reveal the decreased density of corneal subepithelial nerve fiber plexus, activation of stromal cells, increased endothelial cell polymorphism, and decreased cell density. Confocal microscopy might be superior to specular microscopy to analyze the endothelial cells in AACC with corneal edema.

The confocal microscope can directly obtain images of tissues and cells in each layer of the cornea through continuous confocal scanning, detecting various elements of normal and affected corneas in multiple layers and in vivo, without fixation and staining of tissue sections [9–14]. Thus, it is a major method for exploring the pathological mechanism of ophthalmic diseases at the cellular level. In this study, confocal microscopy was used to observe the histological changes for each layer of the cornea in AACC and fellow eyes, which provided a basis and guidance for further exploring the effect of acute ocular hypertension on corneal injury.

Previous studies on corneal injury caused by ocular hypertension have used specular microscopy, which can only observe the number and morphological changes of corneal endothelial cells [7, 8, 18]. Still, the effects of AACC on the changes in corneal subepithelial nerve fibers, stromal layers, and other layers of the tissues remain unclear. The present study showed that acute ocular hypertension in AACC patients not only damaged the corneal endothelial cells but also caused various degrees of pathological changes in other layers of tissues, including corneal epithelial cell swelling, widening of intercellular space, and large vacuoles in epithelial cells in severe cases. The CNFD, CNBD, and CNFL were reduced. The stromal layer was in an activated state, showing swollen stromal cells, enhanced reflection, and cross-linkage into a network.

Previous studies showed that patients with dry eye [16] and diabetes [14, 19] have different degrees of reduced density of the corneal subepithelial nerve fibers, which can even disappear. Roszkowska et al. [13] found that corneal subbasal nerve plexus density reduces with age and myopic refractive error in healthy adults. The reduction in the density of the subepithelial nerve fiber plexus in patients with acute angle closure might be related to corneal dystrophy due to decreased blood flow of the corneal limbal vascular network and corneal epithelium injury caused by acute ocular hypertension [20–22]. The activation of corneal stromal cells suggested an inflammatory response in the corneal tissue. In AACC, the blood-aqueous humor barrier is destroyed, and the anterior segment shows an acute inflammatory response [23]. The expression levels of interleukin (IL)-6, colony-stimulating factor, and vascular endothelial growth factor (VEGF) in the aqueous humor are then increased [24]. Some studies proposed that the increased expression levels of inflammatory factors may result in an AACC event, participating in recurrence [25–27].

Verma et al. [20] found that the density of corneal endothelial cells in PACG patients was not statistically different from that of normal controls. Other studies supported the negative correlation between ocular hypertension and corneal endothelial cells [27–29]. In AACC, long-term ocular hypertension can decrease the density of corneal endothelial cells [29]. The corneal edema is closely related to endothelial cell loss, while in acute angle closure, the corneal edema occurs for the endothelial failure of pomp due to imbalanced imbibition pressure [30]. Sihota et al. [31] found that the average endothelial cell count was 2016 ± 306 cells/mm^2^ in patients with the acute attack lasting < 72 h and 759 ± 94 cells/mm^2^ in those with acute attack lasting > 72 h. This study suggested that compared with the fellow eyes, the polymorphism of corneal endothelial cells in AACC eyes was significantly higher, and the number of cells was significantly lower. In the AACC eyes, endothelial cells could not be analyzed in 17 eyes using specular microscopy due to corneal edema, while confocal microscopy allowed the observation of the morphology and number of endothelial cells in 13 of these 17 eyes. These results indicate that confocal microscopy has the advantages of completing the examination even in the presence of corneal edema, which is a frequent feature of AACC. Using confocal microscopy, the cells were uneven in size, had deposition of punctate high-reflective substances on the cell surface, and the cells were enlarged, deformed, and fused. Furthermore, 10 of the 13 eyes had an endothelial cell count < 1500 cells/mm^2^, with an average of 620 ± 325 cells/mm^2^.

After intravenous infusion of mannitol and oral methazolamide combined with carteolol hydrochloride, brinzolamide, brimonidine tartrate, and pilocarpine nitrate eye drops for IOP-lowering treatment, the average IOP of the 17 severe eyes was 37.2 mmHg, and the average duration of the ocular hypertension event was about 13 days. The results indicated that acute ocular hypertension could damage the corneal endothelial cells, and long-term acute ocular hypertension was critical for the decrease in the number of corneal endothelial cells and abnormal morphology. These results are supported by a rat model of acute ocular hypertension. Indeed, Li et al. [32] observed abnormal morphology and decreased the number of corneal endothelial cells, and these corneal injuries could be reversed after IOP was reduced. Although Verma et al. [20] demonstrated that the differences in the density of corneal endothelial cells at various phases of PACG were not statistically significant; thus, we speculated that an early rapid reduction of IOP could relieve the corneal injury. That will have to be confirmed in future studies.

Both specular microscopy and confocal microscopy can be used for evaluating the corneal endothelial state. Specular microscopy is easy to operate and has been widely used clinically to evaluate the functional status of the corneal endothelial cells, but it cannot be used when corneal edema is present. On the other hand, confocal microscopy can dynamically observe the corneal tissues in real-time, depicting the correlations among various layers of corneal tissues; it is minimally affected by corneal edema, and hence, has gained increasing clinical attention. Therefore, when routine specular microscopy is used for examining the decreased density of corneal endothelial cells, corneal lesions or endothelial cells cannot be analyzed due to corneal edema, and confocal microscopy should be performed for clinical diagnosis and treatment.

There are some limitations to this study. Firstly, the present study was a cross-sectional study, and cause-to-effect relationships cannot be determined. Secondly, no follow-up was performed for patients with corneal edema, and the corneal endothelial cells were not observed in time. Finally, the interval from acute angle-closure attack to initial visit differed in these patients, which have an influence on corneal morphology changes.

## Conclusions

In conclusion, in AACC, the corneal endothelial cells show an abnormal morphology and a decreased density. In addition, the density of the subepithelial nerve fiber plexus was decreased, and the stromal cells were activated, which could be related to the duration of acute ocular hypertension. Such hypertension causes corneal edema, and confocal microscopy can be used to analyze the functional status of the corneal endothelial cells, while specular microscopy is sometimes impossible due to corneal edema.

## Data Availability

The datasets used and/or analyzed during the current study are available from the corresponding author on reasonable request.

## Abbreviations

AACC: Acute angle-closure crisis
PACG: Primary angle-closure glaucoma
IOP: Intraocular pressure
UBM: Ultrasound biomicroscopy
